# Spigelian Hernia Presenting As Abdominal Wall Abscess

**Published:** 2012-04-01

**Authors:** S A Malik, N U Nazeer Kawoosa, B R Zargar

**Affiliations:** 1 Department of General Surgery, Sher-i-Kashmir Institute of Medical Sciences (SKIMS), Soura, Srinagar, Kashmir, 190011, India

**Keywords:** Spigelian, Hernia, Abdominal wall, Abscess

Dear Editor,

Spigelian hernia can be described as a protrusion of a peritoneal sac, organ, or preperitoneal fat through a congenital or acquired defect in the Spigelian fascia, which is the part transeversus abdominis aponeurosis that lies between the semilunar (Spigelian) line and the lateral edge of the rectus muscle, often above the inferior epigastric vessels, at the level of the arcuate line where the fascia is widest and weakest.[1] Some authors have suggested that perforating vessels can weaken the fascia, permitting the entrance of a lipoma and leading to hernia formation.[2] Because of their small neck size, approximately one third of Spigelian hernias in adults appear incarcerated during surgery.

Spigelian hernia is in itself very rare and is difficult to diagnose clinically. It has been estimated that it constitutes 0.12% of abdominal wall hernias.[3] Despite the fact that cases of Spigelian hernia have also been reported in children or even infants, it is a relatively rare hernia that occurs usually in females between 40 and 70 years of age, while the etiologic factors classically associated with this defect are claimed to be obesity, chronic obstructive pulmonary disease, prior surgery, and abdominal trauma.[4] Patients with Spigelian hernia usually complain of pain or lump or both at the site of herniation.[5] The pain is sharp and constant or intermittent, or there is a dragging and uncomfortable feeling.[5] It has been estimated that Spigelian hernias are approximately 2% of the abdominal wall hernias that require emergency operation due to incarceration.[6] When strangulation or incarceration of the herniated contents is present, the pain at the hernia site will be severe and constant. A correct preoperative diagnosis is made in only 53 to 75% of patients, and a significant percentage of incarcerated Spigelian hernias are diagnosed during an emergency laparotomy.[4][7] Spangen reported that 24.1% of Spigelian hernias reach the surgeon incarcerated and that the hernial sac contents are usually found to be small bowel, colon, or omentum.[5] This is supported by our patient in whom hernia sac content was omentum. This report emphasizes possibility of a hernia while a surgeon is dealing with an abdominal wall abscess and the surgeon has to be totally sure about the appropriate treatment to prevent devastating complications.

**Fig. 1:**
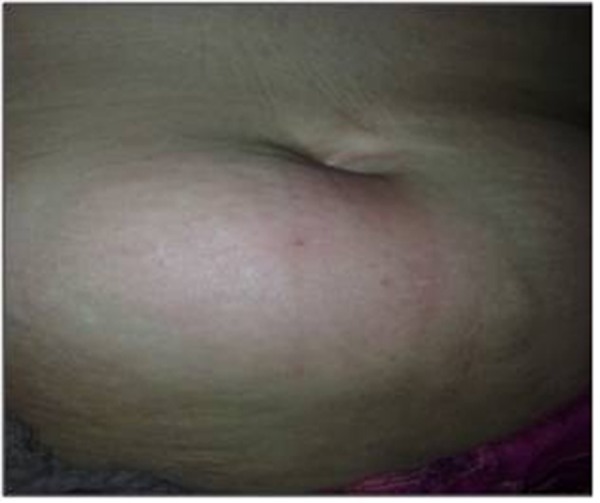
Spigelian hernia as abdominal wall abscess.

**Fig. 2:**
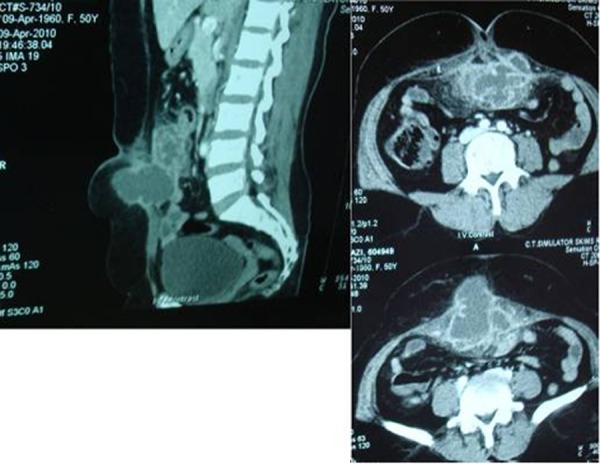
CT scan showing abdominal wall abscess and adjacent bowel walls.

A 60-year old obese female presented to our hospital with history of constant pain and lump in the right lower abdominal wall for the last three days. She also gave history of fever for the last two days. Her bowel habits were normal and did not have any other complaint. On examination, a soft lump of about 4x4x1.5 cm was found on right lower abdomen at the margin of the right semilunar line with inflamed overlying skin (Figure 1 and Figure 2).

**Fig. 3:**
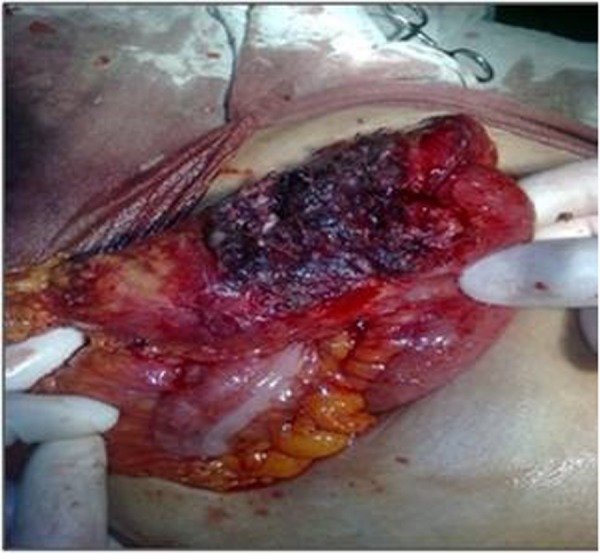
Gangrenous omentum.

There was no cough impulse and lump would not decrease on lying down position. Bowel sounds were normal. Aspiration of swelling revealed purulent materials. Digital rectal examination was normal and x-ray of abdomen revealed nothing significant. Ultrasound (USG) abdomen showed parietal wall abscess with gut loop adherent to it. Computed tomography (CT) scan was advised which confirmed Spigelian hernia with strangulation or incarceration of the omentum and abscess formation in abdominal wall. The patient was taken up for exploratory laparotomy. On exploration, about 25 ml of pus was drained. The defect was seen in peritoneum and gangrenous omentum was seen herniating (Figure 3).

The strangulated omentum was excised and the defect was closed with herniorrhaphy. Pus culture sent grew Klebsiella sensitive to imipenam. The post-operative course of the patient was uneventful. The patient was followed up for over six months without any local recurrence.
